# Applications of Metabolomics in Calcium Metabolism Disorders in Humans

**DOI:** 10.3390/ijms231810407

**Published:** 2022-09-08

**Authors:** Beata Podgórska, Marta Wielogórska-Partyka, Joanna Godzień, Julia Siemińska, Michał Ciborowski, Małgorzata Szelachowska, Adam Krętowski, Katarzyna Siewko

**Affiliations:** 1 Department of Endocrinology, Diabetology and Internal Medicine, Medical University of Bialystok, 15-276 Bialystok, Poland; 2 Clinical Research Centre, Medical University of Bialystok, 15-276 Bialystok, Poland

**Keywords:** metabolomics, hypercalcemia, hypocalcemia, metabolites, parathyroid gland, biomarkers, mass spectrometry

## Abstract

The pathogenesis of the disorders of calcium metabolism is not fully understood. This review discusses the studies in which metabolomics was applied in this area. Indeed, metabolomics could play an essential role in discovering biomarkers and elucidating pathological mechanisms. Despite the limited bibliography, the present review highlights the potential of metabolomics in identifying the biomarkers of some of the most common endocrine disorders, such as primary hyperparathyroidism (PHPT), secondary hyperparathyroidism (SHPT), calcium deficiency, osteoporosis and vitamin D supplementation. Metabolites related to above-mentioned diseorders were grouped into specific classes and mapped into metabolic pathways. Furthermore, disturbed metabolic pathways can open up new directions for the in-depth exploration of the basic mechanisms of these diseases at the molecular level.

## 1. Introduction

Calcium is involved in a multitude of physiological processes, including intracellular signalling, regulation of metabolism, muscle contraction and gene expression [1]. Most calcium in the human body (up to 99%) is found in bones in the form of calcium hydroxyapatite [Ca10(PO4)6(OH)2] and less than 1% of total calcium exists in extracellular fluids. The most active form of calcium in extracellular fluids is the free ionized fraction that directly interacts with calcium channels, calcium-sensing receptors (CaSRs) and cell membranes [2]. The physiological maintenance of calcium levels is mainly regulated by the gastrointestinal tract, bones and kidneys. Parathormone (PTH) is released by the parathyroid gland and regulates calcium within narrow limits by increasing calcium absorption in the intestine, bone-calcium mobilization and calcium reabsorption by the kidneys [3]. Calcium ions are considered to be the principal physiological regulators of PTH secretion via CaSRs [4]. Hence, decreased expression of CaSRs in parathyroid gland tissue is found in patients with hyperparathyroidism and hypercalcemia [5,6]. However, the impact of other factors, including L-amino acids, on CaSRs was also noted [7].

Deviations in the calcium concentration above or below the normal range are now more often diagnosed than in the past, a tendency that mainly occurs due to the increased access to laboratory tests in developed countries. Disorders associated with calcium can be classified as hypocalcemia or hypercalcemia and they usually coexist with other serum biochemical abnormalities, including the levels of phosphate, alkaline phosphatase (ALP), PTH, fibroblast growth factor 23 and vitamin D [8,9].

Hypercalcemia commonly occurs due to malignancy and primary hyperparathyroidism (PHPT). PHPT is a common endocrine neoplastic disorder characterized by the autonomous production of PTH with a broad spectrum of pathophysiological sequelae [10]. It occurs either sporadically or as a familial, hereditary abnormality. The phenotypic diversity of PHPT clinical presentation may be due to the subclasses of parathyroid tumors with distinct molecular profiles [11]. Similarly to PHPT, familial hypocalciuric hypercalcemia (FHH) is a state that also leads to hypercalcemia and the elevation of PTH. Nevertheless, FHH is a rare inherited disorder with a mutation in the CaSRs. The opposite perturbation to hypercalcemia is hypocalcemia, which occurs in vitamin D deficiency and hypoparathyroidism due to irreversible parathyroidectomy, congenital disabilities or radiation damage [12,13].

Changes in calcium levels can induce a wide spectrum of symptoms, including skeletal anomalies, nephrolithiasis and muscle spasms [1]. This frequently affects patients’ quality of life and despite the great pressure on the development of novel methods of diagnosis and treatment, medical approaches to calcium metabolism disorders often pose a significant diagnostic challenge [14]. Functional analyses have become increasingly popular. Among these, metabolomics is the comprehensive analysis of the entire metabolome. This discipline enables the high-throughput analysis of metabolites from cells, tissues, organs and biofluids using modern analytical chemistry techniques [15]. Metabolomics is an important ‘omic’ approach. Metabolites, i.e., small molecules, are markers of various biological processes [1]. The growing popularity of metabolomics and the metabolites’ capacity to serve as sources of novel potential biomarkers used in screening diseases and efficient therapies are observed in various medical disciplines. The metabolome can be explored through either the untargeted analysis of a wide set of unknown metabolites or the targeted quantification of pre-defined known metabolites [16].

Metabolomics is still a relatively new field of science that links cellular phenotypes to their genotypes and delivers biochemical information associated with the regulation of specific gene transcripts [17]. Environmental factors may also induce metabolic changes. Hence, their analysis may help to understand the pathogenesis of diseases and to discover new biomarkers in easy-to-obtain biological samples, including urine and plasma [18]. Cancer-tissue samples can also be studied to indicate in situ metabolic changes [19]. The currently accepted tools used in metabolomics are nuclear magnetic resonance (NMR) spectroscopy and mass spectrometry (MS) combined with various separation methods, including (high-/ultra-pressure) liquid chromatography (LC-MS), gas chromatography (GC-MS) and capillary electrophoresis (CE-MS). The combination of these analytical platforms enables the quantification of low-molecular-weight metabolites [20,21,22]. GC-MS and LC-MS are predominant among the technical platforms for metabolomics, primarily due to their higher sensitivity [23]. However, LC-MS is considered to be the best tool for precise metabolomics, as it is able to quickly separate and identify individual metabolites with the highest throughput of clinical samples and the highest sensitivity [20,21].

Metabolomics has yielded valuable new insights into a number of important biological and physiological processes. In medicine, metabolic profiling can be used to propose novel diagnostic algorithms or to reveal the phenotypic differences in various disorders and then guide personalized therapy [24]. There are still a number of metabolic pathways involved in calcium metabolism diseases that have not yet been identified. Given the versatility and importance of metabolomics, this review covers in details metabolomics research on calcium metabolism disorders in humans. In particular, PHPT, secondary hyperparathyroidism (SHPT), calcium deficiency, osteoporosis and vitamin D supplementation.

## 2. Methods

The studies reported here present the usefulness of a global human metabolic network to interpret plasma metabolites profiling in various branches of medicine [25]. Well-curated metabolomics databases are necessary. This review focuses on metabolomics advances in the disorders of calcium metabolism. We analysed data from publications regarding potential biomarkers and related altered metabolic pathways in human calcium metabolism disorders using metabolomics approaches. PubMed and Scopus databases were searched on 25 April 2022 using the search terms: “metabolomics”, “hyperparathyroidism”, “hypoparathyroidism”, “calcium”, “osteoporosis”, “vitamin D supplementation”, “vitamin D defficiency” and “calcium defficiency” to identify proper publications. Additional search was performed on 22 August 2022. There were no restrictions on publication type, or date. Articles in non-English languages, providing information on gut microbiota, or considering other diseases, not related to human calcium metabolism disorders, were excluded. We reviewed data that might be used to increase the accuracy of calcium metabolism disorders diagnosis and help reveal new therapeutic approaches in humans.

Table 1 summarizes research concerning the biomarkers and related metabolic pathways in human calcium metabolism disorders discovered using metabolomics approaches.

For each disorder, we summarized differentiating metabolites reported in selected publications. These metabolites were classified following the Human Metabolome Data Base (HMDB) classification for polar molecules and Lipid Maps classification for lipidic molecules. This information was used to illustrate which classes of metabolites are involved in different calcium metabolic disorders the most. Moreover, HMDB identifiers for metabolites were retrieved and used for pathways analysis. Pathway analysis was performed using MetaboAnalyst 5.0 (http://www.metaboanalyst.ca/ accessed on 25 August 2022). Only annotated metabolites significantly discriminating studied groups were used for this analysis. The Kyoto Encyclopedia of Genes and Genomes (KEGG) based Homo sapiens library was selected for analysis with a hypergeometric test in over-representation analysis and relative-betweenness centrality in pathway typology analysis (to estimate node importance). Pathway significance was determined from pathway enrichment analysis and based on values for each compound in the dataset.

## 3. Metabolomics in Calcium Metabolism Disorders

Literature revision over the application of metabolomics in calcium metabolism disorders led to the selection of several publications, which were classified into five main categories: PHPT, SHPT, osteopenia and osteoporosis, calcium and vitamin D3 deficiency. 78% of these studies were performed employing untargeted metabolomics and only four publications described the targeted approach. These analyses were performed using various biospecimens. The most frequently used type of the sample was serum (57% of cases). Surprisingly plasma samples were used only in two studies. Urine samples were analyzed in four and parathyroid tissue in two studies. Moreover, only in few studies combined serum samples with urine and fecal samples were analyzed [31,40,41].

Further analysis revealed that 79% of analyses were performed using MS and only 21% employed NMR. This result is not surprising because MS dominated the field of metabolomics due to its outstanding sensitivity. MS usually is used in combination with some separation technique: LC was used in 10 publications, GC and CE in two papers. Surprisingly, there are only two studies where more than one technique was used [26,41]. LC is the most popular separation technique, especially in untargeted studies, because of the metabolite coverage. Changing the chromatographic column type, mobile phases, or modifiers allow separation of different groups of metabolites and therefore provides a broader picture of metabolic changes occurring under given conditions. Here, all LC/MS—based analyses were performed employing RP—chromatography by the use of C18 columns. At this point, it is essential to highlight that all these summaries were performed based on the available information. Unfortunately, a large number of analytical details are not provided in the publications. The lack of even very basic information, unable profound data synthesis and comparison and more importantly, precludes conducting similar studies and reproducing the results. This is particularly important in the case of metabolomics studies, where even minor changes in analytical conditions or data analysis settings can lead to very different results. Therefore, a community should make an effort to report all conditions, even if the profile of a particular journal is not analytical [43].

**Figure 1:**
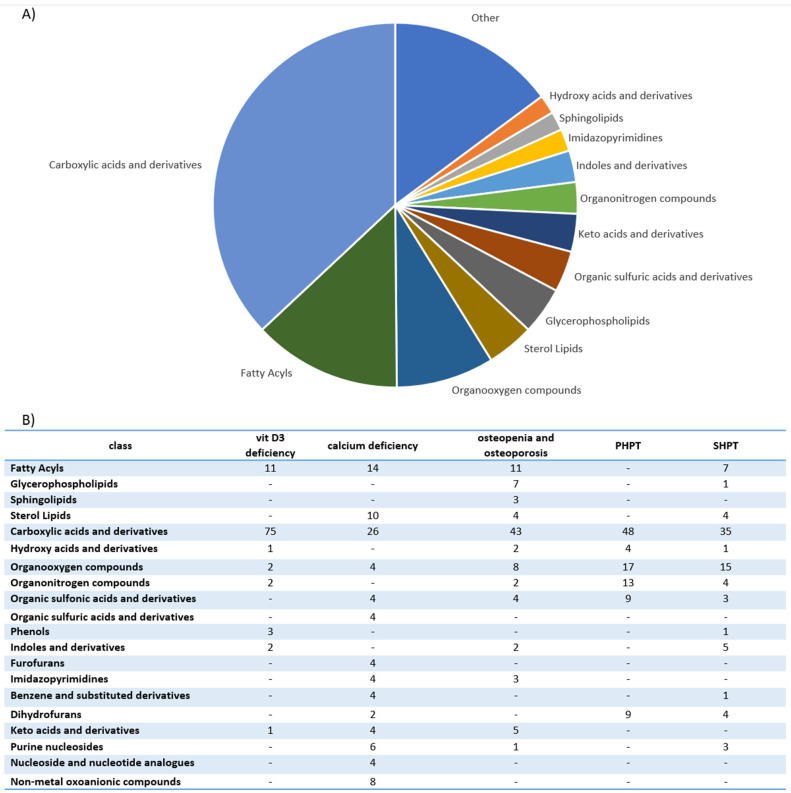
Metabolites altered in calcium metabolism disorders detected in metabolomics studies. (**A**) shows pie chart illustrating distribution of all metabolites across different classes, while (**B**) shows a table with distribution of metabolites across different classes with the stratification for different calcium metabolism disorders. The numbers reflect the number of metabolites assigned to the particular class.

We performed characterization and analysis of all metabolites altered in calcium metabolism disorders (Table S1 in Supplementary Materials). Interestingly, lipidic compounds constitute less than 25% of all metabolites. The largest group of altered metabolites are carboxylic acids and derivatives, represented mostly by amino acids, peptides and analogues (Figure 1A). The second group are fatty acyls with fatty acids and carnitines as the predominant metabolites. Carbohydrates and carbohydrate conjugates constitute the major fraction of the organooxygen compounds, being third largest group. AS can be seen in panel B of Figure 1, the overlap of different metabolite classes between different calcium metabolism disorder is not big. Only carboxylic acids and derivatives and organooxygen compounds are common for all five groups of disorders. This might point to the substantial differences in the metabolic impact caused by each disorder, although all of them are linked with the disruptions in the calcium metabolism.

## 4. Primary Hyperparathyroidism

The excessive secretion of PTH is a common disorder known as hyperparathyroidism. PHPT is one of the most common endocrine diseases in western countries and is caused by an abnormal proliferation of tumor parathyroid cells. Parathyroid tumors are categorized as a genetically heterogeneous group which is characterized by a significant variability in clinical features [44,45]. Both sexes are almost equally affected by hereditary forms of PHPT. However, in sporadic cases, women are much more commonly affected. The increase in the incidence of PHPT is observed among women in their sixth decade of life [46].

Due to advances in diagnostics, the clinical presentation of PHPT evolved in developed countries from a symptomatic to an incidentally discovered and asymptomatic disorder [47]. Recent observational studies suggested that excessive PTH secretion in normocalcemic PHPT, the first subclinical phase of PHPT, might exhibit metabolic disturbances and induce the risks of cardiovascular diseases and tissue dysfunctions [48,49].

Over the last decade, studies provided numerous new insights into the differential metabolic profiles between healthy and pathological parathyroid glands. Palermo et al. in their study, attempted to use Raman spectroscopy to differentiate healthy parathyroid tissue and parathyroid adenomas (PAds). The results demonstrated the strong potential of the Raman spectroscopy method, which showed the different spectroscopic features of these two types of parathyroid tissue. This method was able to correctly classify all subjects with 100% prediction accuracy [50]. Di Masi et al. also aimed to check the metabolic differences between PAds and healthy parathyroid glands. They used Western blotting to assess the expression of specific metabolic enzymes and proteins and a subsequent review of Raman data from the literature. The authors observed increased glucose uptake by the GLUT-1 receptor and an increased level of hexokinase II in PAds compared with normal parathyroid glands. Increased lipid synthesis due to increased levels of ATP-citrate lyase, fatty acid synthase and acetyl-CoA synthetase was also observed in PAds. Moreover, the increased expressions of cytochrome c, glucose-6-phosphate dehydrogenase and 3-phosphoglycerate dehydrogenase were reported in PAds compared with normal tissue. The authors wanted to determine the mechanisms responsible for parathyroid tumorigenesis, which in most cases remain unclear. However, according to the presented results, it was still not obvious if the described metabolic changes were involved in PHPT pathogenesis or rather occurred as a response of the neoplastic remodeling of parathyroid tissue. It is noteworthy that despite the presence of one normocalcemic hyperparathyroidism subject among the nine analyzed female patients, its metabolic profile was comparable to the others [51].

Metabolomics was also used in the study reporting the presence of environmental chemicals in parathyroid tumors (Table 1). Lower levels of organic pollutants were found in normal parathyroid tissue. Polychlorinated biphenyls, dichloro-diphenyl-trichloroethane derivatives and polybrominated diphenyl ethers were detected parathyroid tumors. P,P’-dichlorodiphenyldichloroethylene was present in even 99% of samples. Parathyroid glands may be more susceptible to enrichment of these substances according to high lipid concentration in parathyroid gland tissue or specific sensitivity of receptors in parathyroid cells. Therefore, multiple environmental toxicants may play a role in parathyroid tumorigenesis and further studies are warranted [26].

Battini et al. reported results from the first study checking the differences in tissue metabolomic profiles between a single PAd and multiglandular disease [23]. Multiple-gland disease may occur in PHPT, SHPT and tertiary hyperparathyroidism (THPT). Four-gland hyperplasia occurs in up to 10–15% of patients with PHPT [47]. The method used in their study was (1)H high-resolution magic angle spinning nuclear magnetic resonance ((HRMAS) NMR) spectroscopy. A higher expression of succinate and fumarate, involved in the tricarboxylic acid cycle and elevated levels of myoinositol, scylloinositol, choline, phosphorylcholine and glycerophosphocholine were found in a single adenoma. In contrast, higher levels of antioxidants including glutamate, glutathione and ascorbate were found in a case of multiglandular disease. In the presented study, elevated levels of precursors of phosphatidylinositol (a component of phospholipid membranes) may explain the promising results obtained using 18F-fluorocholine positron emission tomography (PET) in the localization of PAds. First-line therapy in PHPT is surgery and finding all of the affected parathyroid glands poses a huge challenge. Hence, the presented results and their use in intraoperative assessment may have an impact on improving surgical treatment results and on the reduction in surgical costs [23].

According to widespread knowledge, there are currently no studies evaluating plasma metabolite biomarkers related to PHPT. However, their discovery may provide an important new tool for personalizing the clinical management of PHPT. Moreover, a minimally invasive method for diagnosing parathyroid carcinoma (PC) allowing proper treatment to be initiated is very much needed. PC is a rare endocrine malignancy diagnosed in the majority of cases postoperatively upon histological examination due to the lack of reliable clinical diagnostic criteria [52]. A direct approach using metabolomics could be a promising tool to increase the understanding of parathyroid tumorigenesis. 

**Figure 2:**
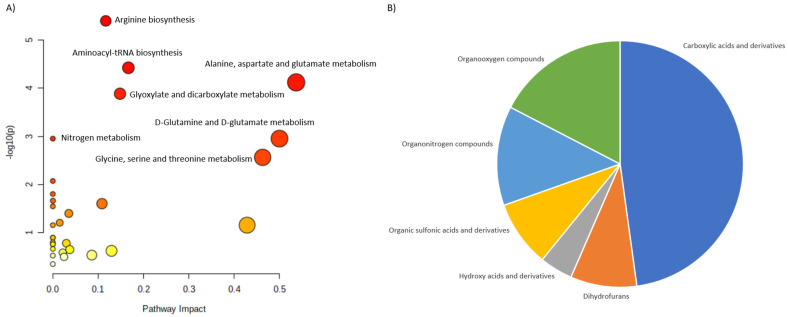
Characterization of metabolites altered in PHPT. (**A**) shows the main pathways, built for the discriminating metabolites reported in review publications. (**B**) shows pie chart, illustrating the distribution of the discriminating metabolites across different metabolite classes.

Based on these reports we selected 24 metabolites, reported as altered in PHPT. 23 of them were assigned to the HMDB identifiers and 21 were matched into the pathways. We classified them into six main categories: Carboxylic acids and derivatives, Dihydrofurans, Hydroxy acids and derivatives, Organic sulfonic acids and derivative, Organonitrogen compounds and Organooxygen compounds (Figure 2).

Among carboxylic acids, amino acids and their derivatives were the predominant metabolites. Organo-oxygen and—nitrogen compounds there the second and third largest groups. Pathway analysis revealed several disturbed pathways, including Arginine biosynthesis, Alanine, aspartate and glutamine metabolism, aminoacyl-tRNS biosynthesis and Glyoxylate and dicarboxylate metabolism among other.

## 5. Secondary Hyperparathyroidism

One of the earliest clinical manifestations of chronic kidney disease (CKD) is SHPT. SHPT occurs due to parathyroid hyperplasia caused by stimuli such as low serum calcium levels associated with renal failure or decreased active vitamin D concentration. The reduction in CaSRs in parathyroid glands is also considered to be one of the pathogenic factors of SHPT [5,6]. The typical serum abnormalities presented by patients with SHPT are hyperphosphatemia, hypocalcemia and elevated PTH [53]. The elevation of PTH occurs due to abnormal calcium homeostasis, decreased glomerular filtration rate and the decline in the metabolically active form of vitamin D, 1,25-dihydroxycholecalciferol (1,25(OH)_2_D)—the main mediator for calcium absorption in the gastrointestinal tract [54,55,56]. In THPT, the enlarged parathyroid gland fails to resolute and continues to oversecrete PTH despite successful kidney transplantation [57].

Serum metabolites correlated with PTH in SHPT were successfully demonstrated in an untargeted metabolomic study performed by Shen involving a group of 35 patients (Table 1). Allyl isothiocyanate, indoleacetaldehyde, L-phenylalanine, D-aspartic acid and D-galactose form a group of biomarkers that might be used to distinguish patients with SHPT from healthy controls with an area under the curve (AUC) of 0.947. Following parathyroid surgery, the concentration of these metabolites restored or tended to be normal [28].

Wu et al. using ultra-performance liquid chromatography-quadrupole time-of-flight mass spectrometry (UPLC-Q-TOF/MS)—based metabolic profiling, identified 32 potential biomarkers for CKD and mineral and bone disorders (Table 1). The authors analyzed metabolites in maintenance-peritoneal-dialysis patients with different levels of PTH. Their results and metabolite concentrations were consistent with Shen’s results, except for downregulated L-phenylalanine in the serum of high-PTH patients. Moreover, dopamine was positively correlated with phosphate concentration and elevated dopamine glucuronide, a promising biomarker for bone disorders in CKD, was noted in the study. Other presented potential biomarkers were the metabolic products of hydroxyproline, reported as serum bone biomarkers, such as glycyl-prolyl-hydroxyproline and glutaminylhydroxyproline. The authors concluded that not only can hyperphosphatemia, hypocalcemia, active vitamin D deficiency and hyperparathyroidism lead to the development of mineral-bone disease, but unidentified metabolic pathways including routes of amino acid metabolism, protein synthesis and steroid hormone metabolism are also involved in this process [27].

L-amino acids in physiological concentrations, especially L-amino acids of the aromatic and aliphatic classes, are known as strong regulators of PTH secretion and thus whole-body calcium metabolism [4]. L-phenylalanine plays the role of allosteric activator of CaSRs, inhibiting PTH secretion [58]. Its increase reported by Shen was considered to be the result of the negative feedback to increased PTH levels. However, Wu’s study did not confirm this finding, presenting a decreased expression in patients with high PTH levels [27]. The inconsistency of this result across studies may be due to the restricted sample sizes and the different types of control groups included in the analyses. Furthermore, the researchers used different methods, therefore the data provided by them may have been hard to compare. Additionally, Shen’s study restricted the impact of nutrition on metabolomic data by collecting blood samples just prior to surgery under severe dietary restrictions [28].

The involvement of D-aspartic acid and L-phenylalanine in abnormal bone metabolism was formerly described. The concentration of these markers may provide new information on the insensitivity of bone turnover in SHPT. Phenylketonuria is a rare inherited disorder associated with phenylalanine hydroxylase deficiency and the accumulation of phenylalanine [59]. Schawahn et al. presented a study investigating the bone mineral density (BMD) of patients with phenylketonuria under dietary treatment using peripheral quantitative computed tomography (CT) to analyze the distal radius. They found minor changes in BMD and the changes were more accentuated within the trabecular-bone compartment [60]. Interestingly, a relationship based on CaSR activation was also demonstrated between phenylalanine and pulmonary hypertension [61]. Moreover, the stimulation of calcitonin release, a single-chain polypeptide hormone secreted by the para-follicular or C cells of the thyroid gland in response to an increase in serum calcium concentration, might be also mediated by elevated plasma L-amino acid levels [58,62].

**Figure 3:**
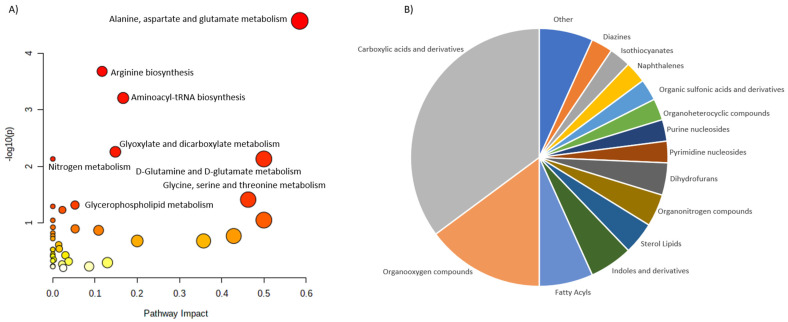
Characterization of metabolites altered in SHPT. (**A**) shows the main pathways, built for the discriminating metabolites reported in review publications. (**B**) shows pie chart, illustrating the distribution of the discriminating metabolites across different metabolite classes.

The literature revision allowed for the collection and characterization of 76 discriminating metabolites. In this case, 74 of them were assign to the HMDB identifiers, but only 67 of the were matched into the pathways. These metabolites were assigned into 16 different classes. Once again, carboxylic acids and derivatives were the largest group followed by the Organooxygen compounds and Fatty acyls (Figure 3).

In contrast to the PHPT, SHPT is characterized by wider group of metabolite classes. This might be related to the differences in the metabolic impact occurring in both disorders, but also to the fact the number of reported altering metabolites was three times higher for SHPT than for PHPT. Larger number of discriminating metabolites covered wider range of metabolite classes. Interestingly, despite differences in the metabolite coverage, pathway analysis revealed highly comparable results. In both cases, Arginine biosynthesis, Alanine, aspartate and glutamine metabolism, aminoacyl-tRNS biosynthesis and Glyoxylate and dicarboxylate metabolism are the most altered pathways.

## 6. Calcium Deficiency

Hypocalcemia is considered to be the main stimulus of PTH synthesis from parathyroid glands [44,63]. Nutritional rickets is still endemic in many countries and occurs due to calcium or vitamin D deficiency. Vitamin D supplementation for all infants and young children significantly reduced the incidence of nutritional rickets in Europe and North America [48].

The first study with urinary metabolic profiling for reliable calcium-deficiency biomarkers was published in 2013 (Table 1). Two biomarkers were selected on the basis of the analyses of calcium-deficient rats according to their significant correlations with calcium intake and they were further assessed in a group of 70 postmenopausal women. The authors concluded that pseudouridine and citrate were useful for population calcium deficiency screening [29].

Wang et al. in their study involving 200 children, investigated urinary metabolomics in patients with nutritional rickets (Table 1). The authors presented 31 differentially expressed proteins. Five selected candidate biomarkers for clinical diagnosis, i.e., sebacic acid, pyrophosphate, citric acid, cAMP and, phosphate were further identified by performing quantitative analyses. A combination of sebacic acid and phosphate was selected as a candidate biomarker with high sensitivity (94.0%) and specificity (71.2%) [30].

Previous metabolomic experiments provided a set of potential diagnostic biomarkers in urine. A serum metabolomic investigation based on UPLC/Q-TOF MS/MS and multivariate statistical analyses to determine whether the biomarkers reported in urine were present in the rats’ serum with a deficiency of calcium. There were 24 calcium deficiency biomarkers performed and revealed. In previous urinary metabolomic studies, three metabolites (taurine, indoxyl sulfate as well as phosphate) have also been found. The levels of phosphoric acid and indoxyl sulfate and were increased in both serum and urine, while the level of taurine was decreased in urine and at the same time it was increased in serum. The AUC combination of any two of them was higher than 0.95 [64]. 

Increased levels of indoxyl sulfate lead to decreased ALP activity, the impaired mineralization of osteoblastic cells and the deregulation of the transcriptional level of collagen type 1. The combination of these changes induces the gradual deterioration of osteogenesis and the impairment of bone turnover common in patients with calcium deficiency [65]. Clinical studies linked elevated levels of indoxyl sulfate to vascular disease, the progression of kidney disease and cognitive impairment [66]. Indoxyl sulfate, phosphoric acid and taurine are involved in processes associated with BMD decline [64]. Taurine serves a variety of functions in bone metabolism, such as osteoclast formation, osteoclast survival and bone-resorption inhibition [67]. Osteoblasts are considered to be direct targets of taurine. They secrete more connective-tissue-growth factor when extracellular-signal-regulated kinase is activated by taurine [68]. An increased level of taurine in serum and its decrease in urine might be explained by the autoregulation processes and the attempt to maintain normal calcium levels by promoting the transport of calcium ions and decreasing taurine renal secretion [64].

**Figure 4:**
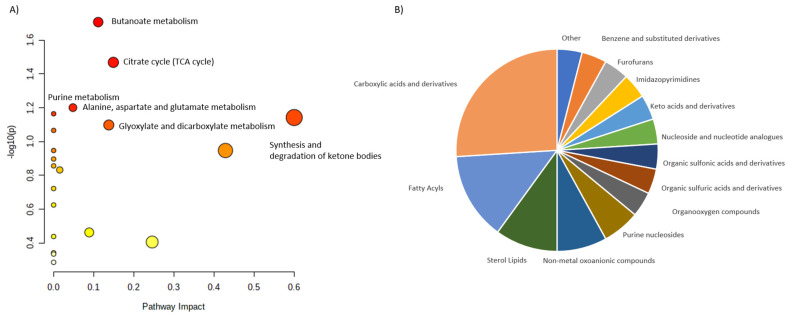
Characterization of metabolites altered in calcium deficiency. (**A**) shows the main pathways, built for the discriminating metabolites reported in review publications. (**B**) shows pie chart, illustrating the distribution of the discriminating metabolites across different metabolite classes.

All these publications cumulatively reported 58 metabolites which level is disturbed in the calcium deficiency. We were able to assign HMDB identifiers to 48 of them and 46 were later matched into pathways. These 58 metabolites were stratified into 15 classes. Carboxylic acids and derivatives with amino acids and derivatives were the largest group. Interestingly, two next largest groups are represented by lipids and are Fatty acyls and Sterol lipids (Figure 4).

In this case there are only few altered pathways with butanoate metabolism, TCA cycle, Purine metabolism and Alanine, aspartate and glutamate metabolism as the most impacted ones. Sterol lipids contributed to the Glyoxylate and dicarboxylate metabolism pathway.

## 7. Osteoporosis

Osteoporosis is characterized by low BMD with the destruction of the bone microstructure. It can lead to increased bone fragility and low-trauma osteoporotic fractures [39]. Osteoporosis in people older than 50 years has a high prevalence rate. It is estimated to be higher (more than 10%) in postmenopausal women [69,70]. Low vitamin D levels as well as together with insufficient calcium intake, estrogen deficiency in postmenopausal women and smoking contribute to osteoporosis [39,71]. The prevalence of osteoporosis and the expenditures associated with its treatment impose a significant socioeconomic burden [72]. In the studies below, the authors tried to implement new approaches to understand the pathogenesis of osteoporosis. Changes in levels of metabolites were found to be associated with altered BMD in humans.

Animal models are frequently used in osteoporosis metabolomics studies. Mao et al. presented the results of a UPLC-QTOF MS analysis indicating that calcium supplementation has beneficial effect on bone loss in ovariectomized rats. The authors showed that calcium supplementation increased the levels of estradiol (E2) and led to changes in metabolites levels, increasing BMD. The administration of higher doses of calcium decreased the levels of bone-turnover markers. The glycerophospholipid metabolic pathway is closely related to calcium supplementation. The main differences in glycerophospholipids metabolism were identified in phosphatidylethanolamines, phosphatidylcholines and ceramides. Additionally, it was found that E2 may affect changes in glycerophospholipids and glycerophospholipid metabolism was considered to be the main potential target pathway of E2 [71]. Zhao used UPLC-Q-TOF-MS-based lipidomics in combination with metabolomics and provided more detailed information about the lipidomic profiles of and alterations in fatty acids, glycerolipids, glycerophospholipids, sphingolipids and sterols in an ovariectomized mouse model of osteoporosis [73]. Wei and colleagues, to investigate the metabolic mechanisms of sarco-osteoporosis, used untargeted UPLC-Q-TOF/MS-based metabolomic profiling and identified 65 differential metabolites in skeletal muscle tissue in 24 mice. According to their results, alterations in several pathways affected by estrogen deficiency were reported. These include glycerophospholipid metabolism, tryptophan metabolism, arginine biosynthesis, histidine metabolism, purine metabolism, thermogenesis and oxidative phosphorylation. With this study, the authors provided new insights into the possible therapeutic effects of estrogens on sarco-osteoporosis [74]. A recent non-targeted metabolomics study presented relationships between kidney, bone and bone marrow of ovariectomized rats with postmenopausal osteoporosis. Selection of organs was strictly connected to their involvement in pathophysiology of postmenopausal osteoporosis. GC-MS was used in metabolic profiling of rats 13 weeks after bilateral ovariectomy or sham surgery. The levels of a methylated amino acids and N-methyl-L-alanine were significantly decreased in kidney, bone and bone marrow of ovariectomized rats. N-methyl-L-alanine, 2-hydroxybutyric acid, (R)-3-hydroxybutyric acid, urea and dodecanoic acid were common differential metabolites in kidney and bone marrow. N-methyl-L-alanine, α-tocopherol and isofucostanol were common differential metabolites between bone and kidney. Glycine, serine and threonine metabolism, tryptophan metabolism, purine metabolism and fatty acid biosynthesis were also disturbed in multiple tissues of ovariectomized rats. In short, the aim of the study was to find metabolic relationships among three selected tissues of ovariectomized rats and analysis of presented metabolites indicated that renal dysfunction, energy metabolism disorders, inhibition of osteoblast activity, activation of osteoclast activation and stimulation of oxidative stress can be induced with postmenopausal effect of bilateral ovariectomy. Kidneys have an impact on bone regeneration by excreting acids and calcium reabsorption, while the oxidative stress status of the bone marrow is related to the growth and development of osteoblasts [75].

Animal models were also used to evaluate efficacy of traditional Chinese herb containing osthole against osteoporosis in ovariectomized rats. In this study, high throughput metabolomics method was applied to discover biomarkers and altered pathways as potential targets. Osthole effectively altered 19 endogenous metabolites involved in several metabolic pathways, including starch and sucrose metabolism, arachidonic acid metabolism and linoleic acid metabolism [76].

Zhang et al. aimed to find BMD-associated markers that are predictive of fracture risk (Table 1). The authors assessed 209 plasma metabolites using LC-MS/MS, measured femoral-neck BMD and lumbar-spine BMD from 2 to 10 years later and assessed osteoporotic fractures at follow-up up to 27 years later. Incorporating the newly identified 27 metabolites presented by the authors significantly improved fracture prediction. The threonine-, serine—and glycine-metabolism pathways (serine, dimethylglycine, glycine and creatine) were significantly enriched. Moreover, glycine, phosphatidylcholine and triacylglycerol were negatively associated with femoral-neck BMD and two of these metabolites, phosphatidylcholine and triacylglycerol, were also negatively associated with lumbar-spine BMD [39]. Similar results were presented in an independent study performed by Zhao et al. (Table 1). Both studies provided novel insights into the pathogenesis of osteoporosis. Moreover, Zhao concluded that metabolic alterations associated with an increased risk for osteoporosis may develop early in life, even in premenopausal women [37,39].

You et al. in their cross-sectional study, identified a group of metabolites for characterizing low BMD in postmenopausal women (Table 1). Among seven selected metabolites, elevated glutamine was associated with low BMD; in contrast, elevated lactate, acetone, lipids and very-low-density lipoprotein protected against low BMD [33]. Metabolites were also found to be good markers for predicting bone loss in postmenopausal women in other investigations (Table 1). Two investigations confirmed significantly decreased serum diglycine and cystine levels in the study groups. In contrast, hydroxyproline, the degradation indicator of collagen type 1, was increased in postmenopausal women with low BMD. Most amino acid levels, except for branched-chain amino acids, were also increased [35,36].

An untargeted MS-based metabolomics technique was used to analyze 69 patients’ serum samples and identify metabolic panel for patients with low bone mineral density (Table 1). The results provided clear separations between patients with low bone mineral density and healthy controls. The most frequently dysregulated metabolic pathways in low bone mineral density included histidine metabolism, glyoxylate, aminoacyl-tRNA biosynthesis, dicarboxylate metabolism and unsaturated fatty acid biosynthesis. Additional analyses revealed differences between patients with osteopenia and osteoporosis, including different levels of carboxy-4-methyl-5-propyl-2-2furanopropionic acid, carnitine derivatives, phosphatidylcholine, sphingomyelin and palmitic acid [32].

The large population-based study conducted by Ling et al. presented evidence connecting the functional composition of the gut microbiota and metabolomics with osteoporosis. The idea of the study was derived from previous smaller studies and limited observations in humans. Fecal and serum metabolomics were applied to show that tryptophan and tyrosine metabolism and isoleucine, leucine and valine degradation were related to the identified microbiota biomarkers and to osteoporosis, respectively [40].

In osteoporosis, the following metabolic pathways are altered: aminoacyl-tRNA biosynthesis; proline and arginine metabolism; valine, leucine and isoleucine biosynthesis; as well as biosynthesis of arginine. Interestingly, similar alterations were found in arthritis [69]. Furthermore, polyunsaturated fatty acid metabolism is impacted in postmenopausal osteoporosis due to the decline in estrogen levels, which contributes to osteoclastogenesis [34,69].

To conclude, despite multiple studies, it is still not possible to precisely assess the risk of potential decreases in BMD in postmenopausal women. The existing investigations yielded contradictory results. The differences between epidemiological and experimental investigations, as well as the confounding factors in sample preparation, detection and data analysis, could explain these discrepancies. Most of the published metabolomics-based studies of osteoporosis so far used untargeted metabolomic techniques and further studies of the identified metabolites are warranted to fully understand their interactions.

**Figure 5:**
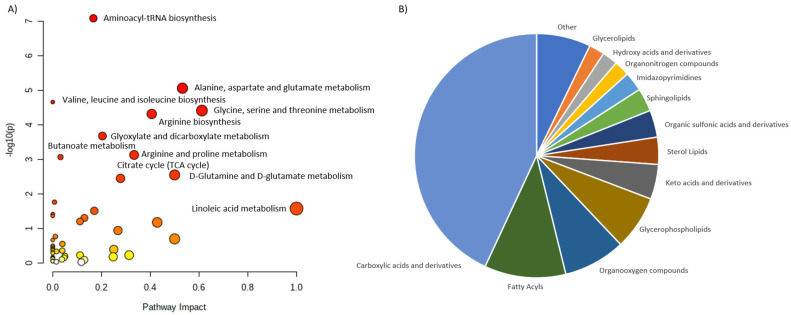
Characterization of metabolites altered in osteopenia and osteoporosis. (**A**) shows the main pathways, built for the discriminating metabolites reported in review publications. (**B**) shows pie chart, illustrating the distribution of the discriminating metabolites across different metabolite classes.

The largest number of publications was reviewed for the osteopenia and osteoporosis, which resulted in the largest number of collected metabolites. We summarized 201 molecules, out of which 188 were assigned to the HMDB identifiers, while pathway analysis was performed for 183 compounds. All these metabolites were distributed across 21 classes. 84 molecules were assigned to the Carboxylic acids and derivatives, 21 to Fatty acyls, 16 to Organooxygen compounds and 14 to Glycerophospholipids (Figure 5).

This large number of differentiating metabolites resulted in the big number of altered pathways. Despite relatively high number of altered lipids, majority of pathways is related to different amino acids metabolism. Similarly to the calcium deficiency, TCA cycle was also matched, however with lower significance and impact.

## 8. Vitamin D Supplementation

There is an ongoing debate on the potential benefits of vitamin D for cardiovascular disease risk, several types of cancers, all-cause mortality and other chronic illnesses. Application of LC-MS/MS in the assay of vitamin D proved that metabolites devoid of biological activity can also serve as disease indicators. Not only 25-OH-D3 or 1,25-(OH)_2_D3 but also other circulating vitamin D metabolites can be detected with this approach. Additional metabolites or their ratios can provide additional diagnostic information. The elevated 25-OH-D_3_/24,25-(OH)_2_D_3_ ratio may help to identify patients with CYP24A1 mutations. Therefore, the differential diagnosis of vitamin D-related hypercalcemia can be supported with analysis of the complete vitamin D metabolome [77]. New members of the vitamin D metabolome were identified. One of them is 20S(OH)D3, a metabolite produced by CYP11A1, with its activity being similar to that of 1α,25(OH)2D3 but with a lack of effects on calcium levels [78].

Spermidine and spermine were found to be involved in the activation of the vitamin D receptor and the induction of spermidine N1-acetyltransferase activity was found to be triggered by 1 alpha,25(OH)2D3 [79,80]. Shirvani et al. presented the results of their study in which they checked the metabolomic responses to varying doses of vitamin D supplementation (Table 1). The authors analyzed the urinary and serum metabolomic profiles before and after six months of supplementation. Their findings were consistent with those of other studies. They demonstrated the alterations in gene expression and metabolomics profiles in response to the same dose of vitamin D3 supplementation. Hence, metabolomics may be used to identify subjects more or less responsive to vitamin D3 [41]. Similar findings were presented in 2010 by Elnenaei et al. (Table 1). They presented disparities between individuals’ responses to vitamin D and calcium supplementation in postmenopausal women with genetic variations in oestrogen receptor 1 gene and vitamin D receptor gene. Furthermore, metabolomic studies revealed distinctive patterns of metabolic profiles of blood and urine that segregated with genotype. NMR studies indicated unique patterns of metabolites, separating non-responders from responders and controls. However, all patients with T-score < −1 were qualified as a group with low bone mass. Patients with osteopenia and osteoporosis were in the same group. Therefore, further studies concerning level of T-score might be interesting [31].

Another more recent study provided new information on direct negative impact of high daily doses of vitamin D supplementation on skeletal muscles (Table 1). Patients treated with 2800 IUs of vitamin D per day presented higher serum levels of creatinine, choline and urea comparing to a control group that receives a placebo. This study was a response to other studies presenting in recent years adverse effects of too high daily doses of vitamin D, including negative effects on risk of falls and muscle strength [42].

Vitamin D2 and vitamin D3 are two main forms with different pharmacokinetic properties used in supplementation. Urinary metabolite profiling by liquid chromatography electrospray ionization quadrupole time-of-flight mass spectrometry (LC-ESI-QTOF-MS) was used to assess the potential biological differences of vitamin D3 (15000 IUs) and D2 (20000 IUs) supplementation in prediabetic patients. Urine samples were taken before supplementation and after 12 weeks of treatment. Nevertheless, no biological differences were found between vitamin D2 and D3 supplementation at levels of circulating 25(OH)D that are equivalent. There were no differences in urinary metabolite profiles [81].

**Figure 6:**
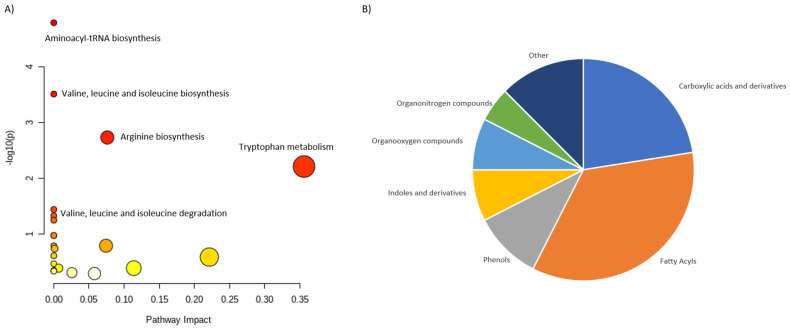
Characterization of metabolites altered in vitamin D3 deficiency. (**A**) shows the main pathways, built for the discriminating metabolites reported in review publications. (**B**) shows pie chart, illustrating the distribution of the discriminating metabolites across different metabolite classes. Second largest group of metabolites are Carboxylic acids and derivatives, followed by Phenols, Indoles and derivatives and Organooxygen compounds. The location of these metabolites in the metabolic pathways showed high alterations in the amino acids’ metabolism. Tryptophan metabolism, Arginine biosynthesis and Valine, leucine and isoleucine biosynthesis together with Amino-tRNS biosynthesis were the most impacted pathways.

We found 42 metabolites reported as altered in the vitamin D3 deficiency. Surprisingly, all 42 molecules were assigned to the HMDB identifiers and further matched into the pathways. These metabolites were distributed across 11 classes. Interestingly, this is the only disorder, where the most predominant group are lipids, in particular Fatty acyls (Figure 6).

## 9. Conclusions

The combination of multiple omic technologies, including transcriptomics, proteomics and metabolomics, is perhaps the next major step towards a full understanding of the pathophysiology of chronic diseases. The applications of metabolomics presented in this review delineate the metabolic profile in calcium metabolism disorders. However, further studies are necessary to combine untargeted metabolomic research with targeted metabolomics to determine the significantly altered metabolites and improve the presented findings. Furthermore, efforts aimed at identifying specific metabolic pathways may serve as targets for treatment. Nevertheless, improved knowledge of the molecular alterations underlying hyperparathyroidism, the identification of PC biomarkers that could allow them to be used as discriminators of this disorder and new tools to assess the risk of fractures and decreases in BMD are undoubtedly desirable objectives. The creation of a vast biomarker library for patients with specific calcium disorders should be the purpose of future studies. To summarize, more in-depth omics studies remain to be performed for the refinement and validation of the molecular profiles described in the present review.

## Figures and Tables

**Table 1:** Biomarkers and related metabolic pathways in human calcium metabolic disorders discovered using metabolomics approaches.

Disorders	Approaches	Study Groups	Sample Type	Metabolite Change	Authors
PHPT SHPT THPT	HRMAS NMR spectroscopy	-18 patients with PHPT; -11 patients with SHPT; -3 patients with THPT.	Parathyroid tissue	Single adenoma and multiglandular disease could be distinguished with metabolomic profiling. Single adenoma: ↑ fumarate, choline, β-glucose, myo-inositol, ascorbate, glycine, scyllo-inositol. Multiglandular disease: ↑ glutamate, glutamine, taurine, aspartate, lactate, GSH. PHPT and SHPT could be distinguished with metabolomic profiling. PHPT: ↑ β-glucose, GSH, ascorbate, myo-inositol glutamate, phosphorylcholine, taurine. SHPT: ↑ fumarate, serine, choline, aspartate, glycerophosphocholine glutamine.	Battini et al., 2016 [23]
PHPT SHPT	GC-MS LC-MS	-40 patients with PHPT; -40 patients with SHPT; -4 patients with normal parathyroid gland.	Parathyroid tissue	Polychlorinated biphenyls, dichloro-diphenyl-trichloroethane derivatives and polybrominated diphenyl ethers were detected in parathyroid tumors. Polychlorinated biphenyl-49 and polychlorinated biphenyl-28 and levels were positively correlated with parathyroid tumor mass. The levels of calcium serum were inversely correlated with concentrations of polybrominated diphenyl ether-47. Polychlorinated biphenyl-49 and p,p’-dichlorodiphenyldichloroethylene were not detected in normal parathyroid gland tissue.	Hu et al., 2021 [26]
SHPT	UPLC-Q- TOF/MS	Maintenance- peritoneal-dialysis patients: -19 disease controls with PTH 150–300 pg/mL; -19 patients with PTH > 300 pg/mL.	Serum	32 unique metabolites were identified in the high-PTH group: ↑ N-(1-Deoxy-1-fructosyl)-tryptophan, N-acetylserotonin glucuronide, dopamine glucuronide, prolyl-tyrosine, glycylprolylhydroxyproline, aminohippuric acid, 2-phenylglycine, 4-carboxyphenylglycine, N-(3-Indolylacetyl)- L-isoleucine, glutaminyl-hydroxyproline, diethyl fumarate, isopropyl citrate, (R)-2-methylmalate, glutamyl-glutamate, N-acetylaspartylglutamic acid. Only 2 of 32 found metabolites were downregulated: ↓ cytidine and L-phenylalanine. Most identified metabolites were not primary metabolites.	Wu et al., 2015 [27]
SHPT	UPLC-MS	-15 patients in the preoperative group with SHPT with PTH level > 600 pg/mL; -15 patients in postoperative group, after parathyroidectomy plus forearm transplantation due to SHPT with PTH level < 150 pg/mL; -5 healthy controls.	Serum	5 metabolites were highly correlated with SHPT. Biomarker group with SHPT: ↑ allyl isothiocyanate, D-aspartic acid, L-phenylalanine. ↓ D-galactose, indoleacetaldehyde. -preoperative group vs. healthy controls (AUC = 0.947); -postoperative group vs. healthy controls (AUC = 0.6).	Shen et al., 2019 [28]
Calcium deficiency	UPLC-Q-TOF MS/MS	Phase I: male rats. Phase II: 70 postmenopausal women.	Urine	Biomarkers (Phase I): glycine, sebacic acid, oxoglutaric acid, pyrophosphoric acid, pseudouridine, taurine, phenylacetylglycine, indoxyl sulfate. 2 biomarkers (pseudouridine and citrate) were further confirmed in 70 women.	Wang et al., 2013 [29]
Nutritional rickets	UPLC−MS/MS	-115 children with rickets; -85 healthy children.	Urine	31 biomarkers of nutritional rickets were identified. 5 candidate biomarkers for clinical diagnosis were screened (phosphate, pyrophosphate, citric acid, cAMP, sebacic acid). The combination of sebacic acid and phosphate was selected as the candidate biomarker with high sensitivity (94.0%) and specificity (71.2%) (AUC = 0.85).	Wang et al., 2014 [30]
Osteopenia and osteoporosis	NMR spectroscopy	56 postmenopausal women: -36 with low bone mass (T-score < −1); -20 with normal bone mass.	Serum and Urine	Disparities between individuals’ responses to vitamin D and calcium supplementation in patients with genetic variations in oestrogen receptor 1 gene and vitamin D receptor gene were found. NMR studies indicated unique patterns of metabolites, separating responders from non-responders and controls.	Elnenaei et al., 2010 [31]
Osteopenia and osteoporosis	LC-MS	69 patients: -25 patients with osteoporosis; -22 patients with osteopenia; -22 patients with normal bone mineral density.	Serum	116 metabolites were associated with low bone mineral density compared to controls (94 metabolites were dysregulated: ↑52, ↑42). The most frequently dysregulated metabolic pathways in low bone mineral density: histidine metabolism, glyoxylate, aminoacyl-tRNA biosynthesis, dicarboxylate metabolism and unsaturated fatty acid biosynthesis. 35 metabolites were dysregulated between patients with osteopenia and osteoporosis: ↑11(3-carboxy-4-methyl-5-propyl-2-2furanopropionic acid, carnitine derivatives) and ↓24(phosphatidylcholine, sphingomyelin, palmitic acid) in patients with osteopenia compared to patients with osteoporosis).	Aleidi et al., 2021 [32]
Osteoporosis	HRMAS NMR spectroscopy	601 healthy Taiwanese women (40–55 years old).	Plasma	7 metabolites characterizing low BMD were identified. Elevated glutamine was significantly associated with low BMD. Elevated lactate, lipids, acetone and very-low-density lipoprotein protected against low BMD. Metabolomic profiling may improve the risk prediction of osteoporosis.	You et al., 2014 [33]
Osteoporosis	GC-MS	364 women: -Premenopausal women with normal BMD; -Postmenopausal women with normal BMD; -Postmenopausal women with osteopenia; -Postmenopausal women with osteoporosis.	Serum	12 metabolites were able to differentiate low-BMD groups from normal-BMD groups. 5 free fatty acids (11,14-eicosadienoic acid, oleic acid, LA and AA) had the greatest potential to be used as osteoporosis biomarkers. ↑ Arachidonic acid, eicosadienoic acid, lysine, linoleic acid, tryptophan, allose, oleic acid. ↓ 3-hydroxy-l-proline, homoserine, pyruvic acid.	Qi et al., 2016 [34]
Osteoporosis	CE-TOFMS	Women (39–64 years old).	Serum	57 metabolites differed significantly among various groups. Diglycine and cystine were lower in the low-BMD group. Hydroxyproline was higher in the low-BMD group. Metabolomic profiling may improve the risk prediction of osteoporosis.	Miyamoto et al., 2017 [35]
Osteoporosis	CE-TOFMS	Women (31–69 years old): -30 premenopausal and normal BMD; -46 postmenopausal and normal BMD; -33 postmenopausal and low BMD.	Serum	52 metabolites differed significantly among various groups. Metabolomic profiling may improve the risk prediction of osteoporosis. Ornithine, arginine, citrulline, creatine and urea levels were increased in postmenopausal groups. The level of guanidinoacetate was decreased in postmenopausal groups. The levels of most amino acids, except branched-chain amino acids, were increased in postmenopausal women with low BMD.	Miyamoto et al., 2018 [36]
Osteoporosis	LC-MS	136 women (20–40 years old): -65 with low hip BMD; -71 with high hip BMD.	Serum	14 metabolites (7 amino acids and amino acid derivatives and 5 lipids: 3 bile acids and 2 organic acids) were associated with a risk of low BMD. Glutamic acid, threonine, taurine, GABA and cysteine were significantly associated with BMD. Metabolomic profiling may improve the risk prediction of osteoporosis.	Zhao et al., 2018 [37]
Osteoporosis	(1)H-NMR	-18 healthy volunteers; -18 diabetic patients with disordered bone metabolism.	Plasma	Metabolomic profiling may improve the risk prediction of diabetic osteoporosis. ↑ Isoleucine valine, glutamine, alanine, inositol, proline, leucine, glucose, 1-methyl-histidine, tyrosine, N-acetylglycoprotein. ↓ O-acetylglycoprotein, creatine, α-ketoglutaric acid, citrate.	Liang et al., 2020 [38]
Osteoporosis	LC-MS/MS	Discovery cohort: 1552 participants. Replication cohort: 634 participants.	Serum	27 metabolites were associated with femoral-neck BMD or lumbar-spine BMD. Glycine, triacylglycerol and phosphatidylcholine were negatively associated with femoral-neck BMD. Phosphatidylcholine and triacylglycerol were negatively associated with lumbar-spine BMD. Authors replicated improvement of fracture prediction with selected metabolites in 634 participants. Metabolomic profiling may improve the risk prediction of osteoporosis.	Zhang et al., 2021 [39]
Osteoporosis	UPLC−MS/MS	971 adults.	Serum and feces	Isoleucine, valine and leucine degradation was associated with osteoporosis. Strong evidence linking gut dysbiosis, fecal metabolomics and serum metabolomics with osteoporosis was reported.	Ling et al., 2021 [40]
Vitamin D3 deficiency	DI-LC/MS/MS GC-MS	30 healthy adults were given 600, 4000 or 10,000 IUs of vitamin D3/day for 6 months.	Serum and urine	Statistically significant changes in 11 metabolites (7 from serum and 4 from urine) after 6 months of vitamin D3 supplementation were found. There was a distinct difference in the targeted metabolites between the more and less vitamin D3 responsive participants. Targeted analysis included 83 metabolites from serum and 36 metabolites from urine.	Shirvani et al., 2020 [41]
Vitamin D3 deficiency	(1)H-NMR	76 postmenopausal women with Vitamin D insufficiency (<50 nmol/l) were given 2800 IUs of vitamin D3/day or placebo for 12 weeks	Serum	Supplementation of vitamin D significantly increased serum levels of carnitine, choline and urea and trimethylamine-N-oxide tendency to rise.	Bislev et al., 2020 [42]

PHPT = primary hyperparathyroidism; SHPT = secondary hyperparathyroidism; THPT = tertiary hyperparathyroidism; AUC = area under the curve; HRMAS= high-resolution magic angle spinning; NMR = nuclear magnetic resonance; UPLC = ultra-performance liquid chromatography; Q-TOF = quadrupole time of flight; MS= mass spectrometry; GC = gas chromatography; CE = capillary electrophoresis; LC = liquid chromatography; DI-LC/MS/MS = direct-flow-injection mass spectrometry; BMD = bone mineral density; (↑) = increased metabolite levels; (↓) = decreased metabolite levels.

## Supplementary Materials

The following supporting information can be downloaded at: https://www.mdpi.com/article/10.3390/ijms231810407/s1.
